# Relationships between sexual violence and chronic disease: a cross-sectional study

**DOI:** 10.1186/1471-2458-14-1286

**Published:** 2014-12-16

**Authors:** Jeanie Santaularia, Monica Johnson, Laurie Hart, Lori Haskett, Ericka Welsh, Babalola Faseru

**Affiliations:** Kansas Department of Health and Environment, Bureau of Health Promotion, Topeka, KS USA; Department of Preventive Medicine and Public Health, University of Kansas Medical Center, 3901 Rainbow Boulevard, MS 1008, Kansas City, KS 66160 USA; Department of Family Medicine, University of Kansas Medical Center, Kansas City, KS USA

**Keywords:** Sexual violence, Chronic disease, Health risks, Behaviors, Mental health, BRFSS

## Abstract

**Background:**

Sexual assault is a traumatic event with potentially devastating lifelong effects on physical and mental health. Research has demonstrated that individuals who experience sexual assault during childhood are more likely to engage in risky behaviors later in life, such as smoking, alcohol and drug use, and disordered eating habits, which may increase the risk of developing a chronic disease. Despite the high prevalence and economic burden of sexual assault, few studies have investigated the associations between sexual violence and chronic health conditions in the US. The purpose of this study is to identify associations between sexual violence and health risk behaviors, chronic health conditions and mental health conditions utilizing population based data in Kansas.

**Methods:**

Secondary analysis was done using data from the 2011 Kansas Behavioral Risk Factor Surveillance System sexual violence module (N = 4,886). Crude and adjusted prevalence rate ratios were computed to examine associations between sexual assault and health risk behaviors, chronic health conditions and mental health conditions, overall and after adjusting for social demographic characteristics. Additional logistic regression models were implemented to examine the association between sexual assault and health risk behaviors with further adjustment for history of anxiety or depression.

**Results:**

There was a significantly higher prevalence of health risk behaviors (heavy drinking, binge drinking and current smoking), chronic health conditions (disability, and current asthma) and mental health conditions (depression, anxiety, and suicidal ideation) among women who ever experienced sexual assault compared to women who did not, even after adjustment for potential confounders.

**Conclusions:**

Study findings highlight the need for chronic disease prevention services for victims of sexual violence. There are important implications for policies and practices related to primary, secondary, and tertiary prevention, as well as collaborations between sexual violence, chronic disease, and health risk behavior programs.

## Background

Sexual assault is a traumatic event with potentially devastating lifelong effects on physical and mental health. According to the Centers for Disease Control and Prevention (CDC), one in four women and one in seven men in the U.S. have reported experiencing sexual violence in their lifetime [1, 2]. Sexual violence is defined as any sex act that is perpetrated against someone’s will. It can be classified into four types: a completed sexual act such as rape, 2), an attempted (but not completed) sex act, abusive sexual contact such as intentional touching without consent), and non-contact sexual abuse such as voyeurism, unwanted exposure to exhibitionism, unwanted exposure to pornography, sexual harassment, and threats of sexual violence) [3]. All types of sexual violence involve victims who do not consent, or who are unable to consent or refuse to allow the act. Although similar to intimate partner violence (IPV), sexual violence may involve a perpetrator who is unknown to the victim and pertains specifically to forcible sex acts [4]. While the economic burden of sexual violence is difficult to quantify due to limited data, the existing research indicates that the costs are substantial. With a total estimated cost of $127 billion a year (excluding the cost of child sexual abuse) [5], rape is the most costly of all violent crimes.

Despite the high prevalence and economic burden of sexual assault, few studies have investigated the associations between sexual violence and chronic health conditions, which are major causes of morbidity and mortality. Seventy percent of U.S. deaths each year are attributed to chronic diseases, with heart disease, cancer, and stroke accounting for more than 50 percent of all deaths [6]. Research has demonstrated that individuals who experience sexual assault during childhood are more likely to engage in risky behaviors later in life, such as smoking, alcohol and drug abuse [7], and disordered eating habits [8], which may increase the risk of developing a chronic disease. Such maladaptive behaviors in this population have been attributed to poor self-esteem, stress, mental illness such as post-traumatic stress disorder [9–11], depression [11, 12]), and sleep disorder [13]. A growing body of research suggests that a child’s development and adult functioning can be profoundly impacted by sexual violence. Nearly half of sexually victimized females were raped before the age of 18, and more than 25 percent of sexually victimized males were raped before the age of 10 [14]. A longitudinal study of sexually victimized girls found that sexual abuse was associated with disruptions in the hypothalamic-pituitary-adrenal axis, which is proposed to result in increased susceptibility to stress later in life [15]. A 2010 meta-analysis supports the hypothesis that childhood sexual abuse is related to higher rates of physical health symptoms, including poor general health, gynecologic pain, cardiopulmonary symptoms, and obesity [16]. Similar pathways linking sexual violence that occurs in adulthood to chronic disease may exist. As part of a large public health survey, Smith & Breiding [17] used data from 25 states/territories that administered the Behavioral Risk Factor Surveillance Survey (BRFSS) sexual violence module in 2005 [17]. The BRFSS is a state-level surveillance system of health-related risk behaviors, chronic health conditions, and use of preventive services among adults 18 years and older. Smith & Breiding reported that non-consensual sex among women and men was associated with smoking, excessive alcohol use, Human Immunodeficiency Virus (HIV) risk factors such as use of intravenous drugs, treated for sexually transmitted infection, prostitution, engaged in anal sex without a condom in the past year, arthritis, asthma, activity limitations, high cholesterol, stroke, and heart disease. Women with a history of sexual violence were more likely to experience a heart attack than women without such a history. Similarly, Black et al. [14] found that sexually victimized women were more likely to have asthma, irritable bowel syndrome, and diabetes, and were more likely to experience chronic pain, frequent headaches, and difficulty sleeping. Another study found that women with a history of sexual assault were more likely to smoke, have high cholesterol and hypertension, and to be obese [8].

The aim of this study was to identify the relationship of sexual violence and chronic disease using current Kansas state-level BRFSS data which includes mental health and additional behavioral risk behaviors that have not been examined previously. The results of this study could support the establishment of public health strategies that implement primary, secondary, and tertiary prevention for chronic disease and sexual violence. In addition, the findings may suggest future avenues of research that will further elucidate this relationship.

## Methods

### Data collection/Study participants

In 2011, the Kansas BRFSS implemented a state-added optional module to assess the prevalence of sexual assault among Kansas adults. Kansas BRFSS is an ongoing, annual, population-based random-digit-dial survey of non-institutionalized adults ages 18 years and older living in a private residence with landline and/or cell phone service in Kansas. The study was approved by the Kansas Department of Health and Environment (KDHE) Institutional Review Board. The survey was administered in English and Spanish. Kansas BRFSS uses a split questionnaire design which consists of a core section and an optional module/state-added module section. Questions in the core section are asked of all respondents. Following the core section, the survey splits into two versions (versions A and B), each of which included different questions asked of approximately half of all respondents. A total of 8,160 respondents were randomly assigned to questionnaire version B of the survey, which included the state-added sexual violence module. Respondents were only asked questions from the sexual violence module if they indicated they were currently in a safe place. Survey interviewers prefaced the sexual violence module questions by defining sexual assault for women as including, “things like putting anything into your vagina, anus, or mouth or making you do these things to them after you said or showed that you didn’t want to. It includes times when you were unable to consent, for example, you were drunk or asleep, or you thought you would be hurt or punished if you refused.” Respondents were then asked “has anyone ever had sex with you after you said or showed that you didn’t want them to or without your consent?” Response options were “yes”, “no”, “don’t know”, and “refused”. Women who answered “yes” to this question were defined as sexual assault victims.

### Measures

Nine dichotomous health risk behaviors/conditions, six dichotomous chronic health conditions and three dichotomous mental health conditions were assessed for their association with sexual assault for this analysis. Among the nine health risk behaviors/conditions, seven assessed unhealthy behaviors, and two assessed health risk conditions. The seven unhealthy behaviors are heavy drinking (having more than one drink per day), binge drinking (having four or more drinks on one occasion), obesity (body mass index greater than or equal to 30 kg/m^2^), current smoking (currently smoking every day or some days), no physical activity (no physical activity during the past thirty days other than their regular job), human immunodeficiency virus (HIV) risk factors (answered ‘yes’ to any of the following: used intravenous drugs, treated for a sexually transmitted disease, given or received money or drugs for sex, or had anal sex without a condom in the past year), and no routine check-up with a doctor in the past year. The two health risk conditions examined were diagnoses of high blood cholesterol or high blood pressure.

Among the six chronic health conditions, women were asked if they had ever been told by a doctor, nurse, or healthcare professional that they had coronary heart disease, diabetes, cancer, stroke or asthma. Those who indicated that they had been diagnosed with asthma were then asked if they currently had asthma. Women were defined as having a disability if they answered “yes” to either of the following questions, “Are you limited in any way in any activities because of physical, mental, or emotional problems?” and “Do you now have any health problem that requires you to use special equipment, such as a cane, a wheelchair, a special bed, or a special telephone? (Include occasional use or use in certain circumstances)”.

Among the three dichotomous mental health conditions, women were asked if they have ever been told by a doctor, nurse, or healthcare professional if they had depression (including depression, major depression, dysthymia, or minor depression) or anxiety (including acute stress disorder, anxiety, generalized anxiety disorder, obsessive-compulsive disorder, panic disorder, phobia, posttraumatic stress disorder, or social anxiety disorder). In addition, women who answered “yes” to, “has there been a time in the past 12 months when you thought of taking your own life?” were defined as having suicide ideation.

### Data analysis

Analyses were completed using weighted survey procedures with SAS 9.3 and SAS callable SUDAAN 11.0.1. The prevalence of sexual assault, health risk behaviors/conditions, chronic health conditions, and mental health conditions among Kansas women with corresponding 95% confidence intervals were calculated overall and for selected social demographic sub-groups. Logistic regression models were fit with health risk behaviors, chronic health, and mental health conditions as dependent variables and sexual assault status as the independent variable. Crude and adjusted prevalence rate ratios (PRR) were computed to examine the association between sexual assault and health risk behaviors, chronic health, and mental health conditions overall and after adjusting for key social demographic characteristics (annual household income, marital status, age, race and health insurance).

Logistic regression models examining the association between sexual assault and chronic health conditions and health risk behaviors further adjusted for history of anxiety or depression since anxiety and depression were identified as potential confounders of the relationship between sexual assault and health risk behaviors/conditions.

## Results

In 2011, approximately 9 percent (95% CI: 7.2%-9.9%) of Kansas women 18 years and older had ever experienced sexual assault. Table 1 shows the percentage of Kansas women who were sexual assault victims by selected demographic characteristics. The prevalence of health risk behaviors, chronic health conditions, and mental health conditions by sexual assault status is demonstrated in Table 2.

**Table 1:** **Prevalence of sexual assault among women**, **by demographic characteristics**, **Kansas BRFSS 2011**

	%	95% CI
**Overall**	8.6	7.2-9.9
**Age Groups**		
18-44 years	9.4	6.7-12.0
45-54 years	11	8.8-13.2
55-64 years	9.4	7.6-11.1
65+ years	3.7	2.7-4.7
Race/Ethnicity*		
White, NH	8.4	6.6-10.1
Black, NH	10.2	4.0-16.4
Other/Multi-Race, NH	11.3	5.9-16.7
Hispanic	7.8	3.3-12.4
**Education**		
Less than high school	4.7	2.2-7.2
High school graduate or G.E.D.**	9.3	6.2-12.5
Some college	9.6	7.0-12.1
College graduate	8	6.2-9.7
**Annual Household Income**		
Less than $15,000	18.5	10.8-26.2
$15,000-$24,999	8.1	5.0-11.3
$25,000-$34,999	6.7	3.6-9.8
$35,000-$49,999	9.2	6.1-12.3
$50,000 or more	6.1	4.8-7.4
**Employment**		
Employed for wages/Self-employed	8.1	6.3-9.8
Homemaker/Student	7.8	4.4-11.2
Out of work	18.9	8.3-29.4
Retired	3.3	2.4-4.3
Unable to work	19.6	13.7-25.5
**Marital Status**		
Divorced/Separated	17.7	13.7-21.7
Married/Member of Unmarried Couple	6.8	5.6-8.0
Never married	9.5	4.4-14.6
Widowed	4.5	2.9-6.1
**Population Density**		
Urban	8.4	6.7-10.0
Semi-urban	11.9	7.3-16.5
Densely-settled rural	8.4	4.9-11.8
Rural	8.4	3.6-13.2
Frontier	3.7	0.5-6.7
**Health Insurance**		
No	12.7	7.0-18.3
Yes	7.9	6.7-9.2

*Prevalence estimates for race and ethnicity were age-adjusted to the U.S. 2000 standard population.

** General Educational Development.

**Table 2:** **Prevalence of health risk behaviors, chronic health conditions and mental health conditions among women, by sexual assault status, Kansas BRFSS 2011**

	Experienced Sexual Assault (n = 378*)		Did Not Experience Sexual Assault (n = 4508*)	
	%	95% CI	%	95% CI
**Health Risk Behaviors**				
Heavy drinking	8.1	1.1-15.0	2.9	2.0-3.7
Binge drinking	16.4	8.7-24.1	10.5	8.2-12.8
Obesity	62.8	53.8-71.9	57.9	55.1-60.7
Current smoking	41.2	32.6-49.8	16.8	14.5-19.0
No physical activity	25.1	18.9-31.2	26.6	24.3-28.9
High cholesterol	49.8	42.0-57.7	37.6	35.4-39.8
Hypertension	27.7	21.7-33.7	29.5	27.6-31.4
HIV risk factors	7.5	0.5-14.5	2.5	1.3-3.7
No doctor check-up	30.3	23.2-37.5	25.5	22.9-28.1
**Chronic Health Conditions**				
Disability	48.7	40.4-57.0	23.5	21.7-25.4
Heart disease	5.3	3.1-7.6	3.8	3.2-4.5
Diabetes	11.1	7.1-15.2	8.7	7.8-9.6
Cancer	13.6	8.4-18.9	8.5	7.4-9.5
Stroke	5	2.2-7.9	2.9	2.3-3.4
Current asthma	22.4	14.6-30.2	9.1	7.5-10.6
**Mental Health Conditions**				
Depression	47.3	28.9-55.7	16.7	15.0-18.5
Anxiety	33.9	26.0-41.7	12.8	10.9-14.7
Suicide ideation	18.1	9.7-26.4	3.4	2.4-4.3

*n = unweighted frequency.

Table 3 shows the crude and adjusted prevalence rate ratios for health risk conditions, chronic health conditions, and mental health conditions for Kansas women who were sexual assault victims compared to those who were not. The prevalence of current asthma, living with a disability, ever having cancer, high cholesterol, HIV risk factors, heavy drinking, current smoking, depression, anxiety and suicide ideation were significantly higher among women who ever experienced sexual assault as compared to women who did not. After adjusting for age, education, race, annual household income, and health insurance status, the prevalence of current asthma (PRR: 2.26, 95% CI 1.59-3.21), ever having a disability (PRR: 2.06, 95% CI 1.72-2.47), heart disease (PRR: 1.74, 95% CI 1.05-2.89), stroke (PRR: 2.24, 95% CI 1.15-4.37), heavy drinking (PRR: 2.88, 95% CI 1.49-5.56), binge drinking (PRR: 1.79, 95% CI 1.23-2.62), high cholesterol (PRR: 1.18, 95% CI 1.01-1.37), current smoking (PRR: 1.82, 95% CI 1.44-2.32), depression (PRR: 2.32, 95% CI 1.87-2.88), anxiety (PRR: 1.9, 95% CI 1.48-2.67), and suicide ideation (PRR: 3.64, 95% CI 2.2-6.0) remained significantly higher among women who ever experienced sexual assault as compared to women who did not.

**Table 3:** Crude and adjusted** associations between health risk behaviors, chronic health conditions, and mental health conditions and sexual assault status among women, Kansas BRFSS 2011

	Crude		Adjusted**	
	PRR	95% CI	PRR**	95% CI
**Health Risk Behaviors**				
Heavy drinking	2.79	1.12-6.86	2.88	1.49-5.56
Binge drinking	1.56	0.93-2.61	1.79	1.23-2.62
Obesity	1.08	0.93-1.26	1.04	0.90-1.20
Current smoking	2.45	1.92-3.14	1.82	1.44-2.32
No physical activity	0.94	0.73-1.22	0.95	0.72-1.25
High cholesterol	1.33	1.12-1.57	1.18	1.01-1.37
Hypertension	0.94	0.75-1.18	0.99	0.84-1.17
HIV risk factors	2.98	1.04-8.54	1.28	0.64-2.55
No doctor check-up	1.19	0.92-1.54	1.13	0.86-1.49
**Chronic Health Conditions**				
Disability	2.07	1.72-2.49	2.06	1.72-2.47
Heart disease	1.39	0.88-2.20	1.74	1.05-2.89
Diabetes	1.28	0.88-1.87	1.23	0.88-1.72
Cancer	1.60	1.07-2.40	1.43	0.91-2.24
Stroke	1.75	0.97-3.17	2.24	1.15-4.37
Current asthma	2.47	1.68-3.64	2.26	1.59-3.21
**Mental Health Conditions**				
Depression	2.83	2.30-3.47	2.32	1.87-2.88
Anxiety	2.65	2.02-3.49	1.90	1.48-2.67
Suicide ideation	5.36	3.11-9.23	3.64	2.20-6.00

PRR = prevalence rate ratio.

**Annual Household Income, Education, Martial Status, Age, Race/ Ethnicity, & Health Insurance.

Table 4 shows prevalence rate ratios for chronic health and mental health conditions with further adjustment for history of anxiety or depression for Kansas women who were sexual assault victims compared to those who were not. After additionally adjusting for ever being diagnosed with anxiety or depression, the prevalence of current asthma, having a disability, heavy drinking, binge drinking, and current smoking remained significantly higher among women who ever experienced sexual assault as compared to women who did not.

**Table 4:** Additional adjusted*** associations between health risk behaviors and sexual assault status among women, Kansas BRFSS 2011

	PRR	95% CI
**Health Risk Behaviors**		
Heavy drinking	2.5	1.33-4.71
Binge drinking	1.62	1.11-2.38
Obesity	0.97	0.82-1.15
Current smoking	1.63	1.27-2.09
No physical activity	0.87	0.65-1.17
High cholesterol	1.09	0.93-1.28
Hypertension	0.92	0.77-1.11
HI risk factors	1.15	0.56-2.39
No doctor check-up	1.15	0.87-1.51
**Chronic Health Conditions**		
Disability	1.77	1.47-2.13
Heart disease	1.41	0.80-2.49
Diabetes	1.09	0.77-1.55
Cancer	1.26	0.80-1.99
Stroke	1.91	0.96-3.82
Current asthma	1.73	1.21-2.49
PRR = prevalence rate ratio.		

***Annual Household Income, Education, Martial Status, Age, Race/ Ethnicity, Health Insurance, Anxiety, and Depression.

## Discussions

The prevalence of sexual violence among women in Kansas was nearly nine percent, which was lower than the 18.5 percent prevalence reported in a multistate survey [17]. This difference may be explained by the fact that the multi-state survey measured both attempted and completed sexual violence, while this study only measured completed sexual assault. The current study demonstrated that a history of sexual violence was more common among certain demographic groups, including women who were divorced or separated, women with an income less than $15,000, women who were unable to work, women younger than 65 years of age, and women who lived in a semi-urban area. This is consistent with data from the Bureau of Justice Statistics that found that women who were younger, divorced or separated, and lived in low-income households were most likely to experience sexual assault [18]. The authors found no significant differences in history of sexual assault by education level, health insurance status, or race/ethnicity.

Study findings indicate that sexual violence is linked to several adverse health behaviors, chronic health conditions and mental health conditions, even after adjusting for demographic characteristics. These findings support previous reports linking sexual victimization, risk behaviors, and long-term health conditions [7–9, 16, 17]. Unlike one previous study, the association between HIV risk factors and sexual assault history was not significant after adjusting for demographic characteristics [17].

Given the limitations of the cross-sectional study design, there was no way to establish causal or temporal relationships between sexual violence, demographic characteristics, and health behaviors and conditions. For example, men and women with physical and mental disabilities are three to four times more likely to be sexually assaulted than individuals without a disability [19, 20]. The relationship between alcohol use and sexual assault is also complex; excessive drinking has been found to be a significant cause and consequence of sexual assault [7, 21, 22]. While the association between excessive drinking and sexual assault is well known, alcohol can be a contributing factor in all types of interpersonal violence (i.e., intimate partner violence, assault, homicide) [23, 24].

While the mechanism by which sexual violence, demographic characteristics, and health behaviors and conditions are related remains to be elucidated, it is important to note that sexual violence is overwhelmingly experienced by younger women (80 percent of victims reported that they were raped before 25 years of age [14]) while chronic health problems typically arise later in life. Therefore, sexual victimization likely preceded health conditions for most respondents. One possible explanation for the relationship is that sexual violence causes serious psychological distress, such as post-traumatic stress disorder (PTSD) and depression, which is linked to high-risk behaviors. For example, a 2008 study of college-aged women found that PTSD symptoms mediated the relationship between sexual assault and adverse health events [10]. A history of childhood sexual abuse has been associated with persistent, chronic major depression [25].

Another potential explanation for the relationship between sexual assault and chronic disease is that trauma negatively impacts the body’s regulatory and immune functioning. Black et al. [14] reported that women who were sexually victimized were more likely to experience stress and difficulty sleeping. Insufficient sleep has been linked to numerous chronic conditions, including diabetes [26] and obesity [27]. It has been proposed that insufficient sleep adversely affects the function of the hypothalamus, which regulates appetite and the expenditure of energy [27].

This study is not without limitations. This study has no direct comparisons to other studies because the authors used an abbreviated form of the sexual violence module of BRFSS. Due to the limitations of survey questions, the authors could not evaluate specific details (e.g., co-occurring abuse, at what age a victim had experienced the sexual assault, the nature of the sexual assault, whether or not a victim experienced multiple incidents of sexual assault) that might influence the long-term health outcomes of sexual violence.

Another limitation of the study is that the variables were assessed via self-report and were not verified by medical records, which may lead to underreporting. However, sexual assault is likely also underreported in the medical record, since many incidents of sexual violence go unreported and unrecognized [28]. In addition, the 2011 BRFSS was a telephone survey of only residential households, therefore people with a cell phone or without a landline telephone were excluded from state-added sexual violence module arm of the study. Individuals who only have cell phones are more likely to be younger, have lower incomes, and report binge drinking [29]. These demographic factors are associated with increased prevalence of sexual violence [1, 14, 17]. Because cell phone respondents were not included in the sample, this study likely underreports the prevalence of sexual violence among women in Kansas.

This is the first study to describe the health behaviors and conditions of women in Kansas who have ever experienced sexual violence. It is essential to include questions about sexual violence on future iterations of the BRFSS in order to track changes in the prevalence of sexual violence over time and associations with health behaviors and conditions. Due to the cross-sectional study design in the current study, future longitudinal studies are needed to demonstrate temporality between these factors.

The detrimental effects of sexual violence on victims and society cannot be overstated. Many sexually victimized individuals experience lifelong psychological and physical hardships. Therefore, the findings from this study have important implications for policies and practices related to primary, secondary, and tertiary prevention, and provides further evidence that it is critical to change the social paradigm that supports sexual violence. While more research is needed to determine the cost-benefit of universal screening for sexual assault, it is crucial that healthcare providers are trained in sexual violence and sexual violence management and that they are made aware of the associated health risk behaviors and conditions among victims of sexual assault so that they can take proactive, preventive measures. As recommended by the World Health Organization’s report on Responding to Intimate Partner Violence and Sexual Violence Against Women [30] healthcare providers can listen to survivors of sexual assault without pressure for a response to disclose information, offering comfort to help alleviate or reduce anxiety, and provide written information on coping strategies for dealing with severe stress.

## Conclusions

Associations between sexual assault and chronic disease in Kansas emphasize the need to focus not only on the physical and psychological health consequences related to victimization, but also on potential chronic disease consequences and the overall impact of sexual violence on the health care system. In addition, results from this study provide rationale for collaborations between sexual violence, chronic disease, and health risk behaviors programs to develop prevention and intervention strategies that address this important public health problem.

## Abbreviations

BRFSS: Behavioral risk factor surveillance system
CDC: Centers for disease prevention and control
HIV: Human immunodeficiency virus
IPV: Intimate partner violence
PRR: Prevalence rate ratios
PTSD: Post traumatic stress disorder.
